# Supplementary material for: ProteinShader: illustrative rendering of macromolecules
Source: BMC Struct Biol. 2009 Mar 30;9:19. doi: 10.1186/1472-6807-9-19 (PMC2672931; doi:10.1186/1472-6807-9-19)
Supplement: Additional file 1 — ProteinShader program without source code. This compressed file contains the complete ProteinShader program including associated libraries, but no source code. A README.txt file gives an overview of the ProteinShader distribution, and the index.html file in the help subdirectory has directions on getting started with the program as well as a set of tutorials. [file 1472-6807-9-19-S1.zip › ProteinShader-beta-0_9_4-binary/help/api/org/proteinshader/structure/exceptions/class-use/ColorOutOfRangeException.html]

Uses of Class org.proteinshader.structure.exceptions.ColorOutOfRangeException (ProteinShader API)


|  |  |  |
| --- | --- | --- |
| **Overview** \| **Package** \| **Class** \| **Use** \| **Tree** \| **Deprecated** \| **Index** \| **Help** | |  |
| PREV   NEXT | **FRAMES**    **NO FRAMES**     **All Classes** |  |


---


## **Uses of Class org.proteinshader.structure.exceptions.ColorOutOfRangeException**

| Packages that use ColorOutOfRangeException | |
| --- | --- |
| **org.proteinshader.gui.components.controlpanel** | Holds the JPanels used in the right-side retractable control panel of the GUI. |
| **org.proteinshader.structure** | Holds the classes that store information from a Protein Data Bank file: Structure, Model, Chain, AminoAcid, Heterogen, Water, Atom, Bond, Helix, BetaStrand, Loop, *etc*. |
| **org.proteinshader.structure.visitor.modifiers** | Holds helper classes for the ModifierVisitor class, which is used to modify Drawable objects (Atoms, Bonds, and Segments). |

Uses of ColorOutOfRangeException in org.proteinshader.gui.components.controlpanel

| Methods in org.proteinshader.gui.components.controlpanel that throw ColorOutOfRangeException | |
| --- | --- |
| `abstract  void` | `ColorPanel.modifyGlobal(float red, float green, float blue)`             Prepares a DrawableModifier to modify the RGB color of Drawable objects, and then calls on the modifyGlobal() method of the RadioPanel. |
| `void` | `AtomColorPanel.modifyGlobal(float red, float green, float blue)`             Prepares the AtomModifier to modify the RGB color of Atoms, and then calls on the modifyGlobal() method of the RadioPanel. |
| `void` | `CartoonColorPanel.modifyGlobal(float red, float green, float blue)`             Prepares the SegmentModifier to modify the RGB color of Segments, and then calls on the modifyGlobal() method of the RadioPanel. |
| `abstract  void` | `VisibilityPanel.modifyGlobal(VisibilityEnum visibility, float alpha)`             This helper method for applyVisibility() sets up whatever DrawableModifiers are needed and then calls on the modifyGlobal() method of the radio panel. |
| `void` | `CartoonVisibilityPanel.modifyGlobal(VisibilityEnum visibility, float alpha)`             This helper method for applyVisibility() sets up the the SegmentModifier and then calls on modifyGlobal() method of the radio panel. |
| `void` | `AtomVisibilityPanel.modifyGlobal(VisibilityEnum visibility, float alpha)`             This helper method for applyVisibility() sets up the the AtomModifier and then calls on modifyGlobal() method of the radio panel. |
| `abstract  void` | `ColorPanel.modifyHelices(float red, float green, float blue)`             Prepares a DrawableModifier to modify the RGB color of Drawable objects, and then calls on the modifyHelices() method of the RadioPanel. |
| `void` | `AtomColorPanel.modifyHelices(float red, float green, float blue)`             Prepares the the AtomModifier to modify the RGB color of Atoms, and then calls on the modifyHelices() method of the RadioPanel. |
| `void` | `CartoonColorPanel.modifyHelices(float red, float green, float blue)`             Prepares the the SegmentModifier to modify the RGB color of Segments, and then calls on the modifyHelices() method of the RadioPanel. |
| `abstract  void` | `VisibilityPanel.modifyHelices(VisibilityEnum visibility, float alpha)`             This helper method for applyVisibility() sets up whatever DrawableModifiers are needed and then calls on the modifyHelices() method of the radio panel. |
| `void` | `CartoonVisibilityPanel.modifyHelices(VisibilityEnum visibility, float alpha)`             This helper method for applyVisibility() sets up a SegmentModifier and then calls on modifyHelices() method of the radio panel. |
| `void` | `AtomVisibilityPanel.modifyHelices(VisibilityEnum visibility, float alpha)`             This helper method for applyVisibility() sets up an AtomModifier and a BondModifier, and then calls on modifyHelices() method of the radio panel. |
| `abstract  void` | `ColorPanel.modifyLoops(float red, float green, float blue)`             Prepares a DrawableModifier to modify the RGB color of Drawable objects, and then calls on the modifyLoops() method of the RadioPanel. |
| `void` | `AtomColorPanel.modifyLoops(float red, float green, float blue)`             Prepares the AtomModifier to modify the RGB color of Atoms, and then calls on the modifyLoops() method of the RadioPanel. |
| `void` | `CartoonColorPanel.modifyLoops(float red, float green, float blue)`             Prepares the SegmentModifier to modify the RGB color of Segments, and then calls on the modifyLoops() method of the RadioPanel. |
| `abstract  void` | `VisibilityPanel.modifyLoops(VisibilityEnum visibility, float alpha)`             This helper method for applyVisibility() sets up whatever DrawableModifiers are needed and then calls on the modifyLoops() method of the radio panel. |
| `void` | `CartoonVisibilityPanel.modifyLoops(VisibilityEnum visibility, float alpha)`             This helper method for applyVisibility() sets up a SegmentModifier and then calls on the modifyLoops() method of the radio panel. |
| `void` | `AtomVisibilityPanel.modifyLoops(VisibilityEnum visibility, float alpha)`             This helper method for applyVisibility() sets up an AtomModifier and then calls on modifyLoops() method of the radio panel. |
| `abstract  void` | `ColorPanel.modifySelected(float red, float green, float blue)`             Prepares a DrawableModifier to modify the RGB color of Drawable objects, and then calls on the modifySelected() method of the RadioPanel. |
| `void` | `AtomColorPanel.modifySelected(float red, float green, float blue)`             Prepares the AtomModifier to modify the RGB color of Atoms, and then calls on the modifySelected() method of the RadioPanel. |
| `void` | `CartoonColorPanel.modifySelected(float red, float green, float blue)`             Prepares the SegmentModifier to modify the RGB color of Segments, and then calls on the modifySelected() method of the RadioPanel. |
| `abstract  void` | `VisibilityPanel.modifySelected(VisibilityEnum visibility, float alpha)`             This helper method for applyVisibility() sets up whatever DrawableModifiers are needed and then calls on the modifySelected() method of the radio panel. |
| `void` | `CartoonVisibilityPanel.modifySelected(VisibilityEnum visibility, float alpha)`             This helper method for applyVisibility() sets up the SegmentModifier and then calls on the modifySelected() method of the radio panel. |
| `void` | `AtomVisibilityPanel.modifySelected(VisibilityEnum visibility, float alpha)`             This helper method for applyVisibility() sets up the AtomModifier and the BondModifier and then calls on the modifySelected() method of the radio panel. |
| `abstract  void` | `ColorPanel.modifyStrands(float red, float green, float blue)`             Prepares a DrawableModifier to modify the RGB color of Drawable objects, and then calls on the modifyStrands() method of the RadioPanel. |
| `void` | `AtomColorPanel.modifyStrands(float red, float green, float blue)`             Prepares the AtomModifier to modify the RGB color of Atoms, and then calls on the modifyStrands() method of the RadioPanel. |
| `void` | `CartoonColorPanel.modifyStrands(float red, float green, float blue)`             Prepares the SegmentModifier to modify the RGB color of Segments, and then calls on the modifyStrands() method of the RadioPanel. |
| `abstract  void` | `VisibilityPanel.modifyStrands(VisibilityEnum visibility, float alpha)`             This helper method for applyVisibility() sets up whatever DrawableModifiers are needed and then calls on the modifyStrands() method of the radio panel. |
| `void` | `CartoonVisibilityPanel.modifyStrands(VisibilityEnum visibility, float alpha)`             This helper method for applyVisibility() sets up a SegmentModifier and then calls on the modifyStrands() method of the radio panel. |
| `void` | `AtomVisibilityPanel.modifyStrands(VisibilityEnum visibility, float alpha)`             This helper method for applyVisibility() sets up an AtomModifier and then calls on modifyStrands() method of the radio panel. |
| `void` | `AtomColorPanel.setColor(float red, float green, float blue)`             Clears the Atom modifier and sets the RGB colors. |
| `void` | `CartoonColorPanel.setColor(float red, float green, float blue)`             Clears the SegmentModifier and sets the RGB colors. |

Uses of ColorOutOfRangeException in org.proteinshader.structure

| Methods in org.proteinshader.structure that throw ColorOutOfRangeException | |
| --- | --- |
| `void` | `Drawable.setAlpha(float alpha)`             Sets the alpha component of the RGBA color. |
| `void` | `Drawable.setColor(float[] color)`             Sets the RGBA color by copying the values from the color array given as an argument. |
| `void` | `Drawable.setColor(float red, float green, float blue)`             Sets the (red, green, blue) components of the RGBA color. |
| `void` | `Drawable.setColor(float red, float green, float blue, float alpha)`             Sets the (red, green, blue, alpha ) components of the RGBA color. |
| `void` | `Region.setSegmentAlpha(float alpha)`             Sets the alpha component of the RGBA color for all Segments held by this Region. |
| `void` | `Region.setSegmentRGB(float red, float green, float blue)`             Sets the RGB color on all Segments held by this Region. |

Constructors in org.proteinshader.structure that throw ColorOutOfRangeException

`Drawable(double x,
double y,
double z,
VisibilityEnum visibility,
DrawableEnum drawableType,
float red,
float green,
float blue,
float alpha)`
  
          Constructor for use by subclasses.

Uses of ColorOutOfRangeException in org.proteinshader.structure.visitor.modifiers

| Methods in org.proteinshader.structure.visitor.modifiers that throw ColorOutOfRangeException | |
| --- | --- |
| `void` | `AtomModifier.modify(Atom atom)`             Modifies the ball radius if requested, and then calls on modify() of the superclass, DrawableModifier, to apply any other requested modifications such as visibility, color, or radius for space-filling model. |
| `void` | `DrawableModifier.modify(Drawable d)`             Modifies the visibility, RGBA color, and/or radius of the Drawable object given as an argument. |
| `void` | `SegmentModifier.modify(Segment segment)`             Sets the patterns texture, halftoning texture, Segment color, or decoration type as requested by SegmentModifier methods, and then calls on modify() of the DrawableModifier superclass. |
| `void` | `DrawableModifier.setAlpha(float alpha)`             Stores a memory that the alpha component of the RGBA color needs to be set to the value given as an argument. |
| `void` | `AtomModifier.setColor(float red, float green, float blue)`             Stores a memory that the (red, green, blue) components of the RGBA color need to be set to the values given as arguments. |
| `void` | `SegmentModifier.setColor(float red, float green, float blue)`             Stores a memory that the (red, green, blue) components of the RGBA color need to be set to the values given as arguments. |
| `void` | `DrawableModifier.setColor(float red, float green, float blue)`             Stores a memory that the (red, green, blue) components of the RGBA color need to be set to the values given as arguments. |
| `void` | `DrawableModifier.setColor(float red, float green, float blue, float alpha)`             Stores a memory that the (red, green, blue, alpha) components of the RGBA color all need to be set to the values given as arguments. |

---


|  |  |  |
| --- | --- | --- |
| **Overview** \| **Package** \| **Class** \| **Use** \| **Tree** \| **Deprecated** \| **Index** \| **Help** | |  |
| PREV   NEXT | **FRAMES**    **NO FRAMES**     **All Classes** |  |


---

# *Copyright © 2007-2008*
